# Changes in Substance Abuse and HIV Risk Behaviors over 12-Month Methadone Maintenance Treatment among Vietnamese Patients in Mountainous Provinces

**DOI:** 10.3390/ijerph16132422

**Published:** 2019-07-08

**Authors:** Bach Xuan Tran, Mercy Fleming, Tam Minh Thi Nguyen, Giang Thu Vu, Quan Hoang Vuong, Manh-Tung Ho, Nhue Van Dam, Thu-Trang Vuong, Ha Ngoc Do, Linh Phuong Doan, Carl Latkin, Cyrus SH Ho, Roger CM Ho

**Affiliations:** 1 Institute for Preventive Medicine and Public Health, Hanoi Medical University, Hanoi 100000, Vietnam; 2 Bloomberg School of Public Health, Johns Hopkins University, Baltimore, MD 21218, USA; 3 Vietnam Young Physician Association, Hanoi 100000, Vietnam; 4 School of Medicine and Medical Science, University College Dublin, Belfield, Dublin 4, Ireland; 5 Vietnam Authority of HIV/AIDS Control, Ministry of Health, Hanoi 100000, Vietnam; 6 Center of Excellence in Evidence-based Medicine, Nguyen Tat Thanh University, Ho Chi Minh City 70000, Vietnam; 7 Centre of Interdisciplinary Social Research, Phenikaa University, Yen Nghia, Ha Dong, Hanoi 100803, Vietnam; 8 Faculty of Economics and Finance, Phenikaa University, Yen Nghia, Ha Dong, Hanoi 100803, Vietnam; 9 Faculty of Graduate Studies, National Economics University, Hanoi 100000, Vietnam; 10 Sciences Po Paris, Campus de Dijon, Dijon 21000, France; 11 Youth Research Institute, Ho Chi Minh Communist Youth Union, Hanoi 100000, Vietnam; 12 Center of Excellence in Behavioral Medicine, Nguyen Tat Thanh University, Ho Chi Minh City 70000, Vietnam; 13 Department of Psychological Medicine, National University Hospital, Singapore 119074, Singapore; 14 Department of Psychological Medicine, Yong Loo Lin School of Medicine, National University of Singapore, Singapore 119078, Singapore; 15 Department of Functional Near Infrared Spectroscopy, Department at the Institute for Health Innovation and Technology (iHealthtech), National University of Singapore, Singapore 119078, Singapore

**Keywords:** methadone maintenance, HIV, risk behavior, mountainous Vietnam, substance abuse

## Abstract

Methadone maintenance treatment (MMT) programs have been used worldwide to reduce the number of drug users and for HIV prevention; however, evidence of their effectiveness in mountainous areas is limited. This study aimed to identify changes in substance abuse and sexual practices among MMT patients after treatment in three Vietnamese mountainous provinces. A survey on risk behaviors was conducted among 300 drug users in six MMT clinics prior to and following one year of MMT. Cramér’s effect size of changes was extrapolated to justify the magnitude of the intervention’s effectiveness. A generalized estimation equation was used to find the factors associated with respondents’ substance use and sexual risk behavior. While drug-related risk behaviors were significantly reduced, alcohol and sex-related behaviors remained risk factors for HIV in this group. Additionally, condom use was common among participants at both time points, but not among those having sex with sex workers. Socio-economic characteristics of ethnic, education, occupation, as well as drug use history influenced the possibility of engaging in drug use and/or sexual risk behavior following treatment. Further emphasis on managing these among MMT patients is required, potentially by providing integrated services including smoking and drinking counseling and condom use promotion in accordance with MMT.

## 1. Introduction

HIV remains a significant global health burden, with 36.7 million people estimated to be HIV-positive in 2016, with over 2.1 million new infections reported globally in 2015 alone [1]. Approximately 260,000 of these HIV-positive patients are living in Vietnam [2,3,4], though the new infection rate has stabilized in the country since 2012 [1,5]. It is important to reduce the prevalence of HIV by decreasing the new infection rate through risk prevention and decreased transmission. People who inject drugs are one of the most-at-risk groups of contracting HIV globally [6]; therefore, minimizing risk behaviors among drug users is an important strategy in managing HIV transmission.

The effect of methadone maintenance treatment (MMT) on risk behaviors towards HIV/AIDS has been demonstrated in many studies. In China, Wang et al. showed that those engaged in drug- and sex-related risk behaviors including needle-sharing and unprotected sex were significantly less likely to have received MMT [7], while Chen et al. found MMT adherence to be a significant predictor of lower HIV-related risk behaviors [8]. Longitudinal studies in the US and Canada have also illustrated that MMT programs can have positive sex-related and drug-related outcomes, with this type of intervention being incredibly useful for decreasing HIV risks [9,10,11]. Similar findings have been reported by a number of studies in Vietnam; for instance, a study by Tran et al. in 2012 found that, through facilitating successful antiretroviral therapy, MMT enhanced the health-related quality of life of drug users [12], while another study published in 2013 indicated the influence of MMT not only on improving HIV/AIDS intervention outcomes but also in lightening the economic burden suffered by HIV-infected patients and their families, by reducing their health care service utilization and out-of-pocket (OOP) payment [13].

As it is estimated that up to 30% of HIV-positive patients in Vietnam are people who inject drugs (PWID) [14], the link between MMT and HIV services in Vietnam is particularly strong. A number of studies have evidently proved the beneficial impact of MMT on combating HIV. A longitudinal study conducted in 2009 found that the quality of life of HIV/AIDS patients was improved following MMT [15], whereas another study combining retrospective and prospective non-randomized cohort studies on center-based compulsory rehabilitation treatment (CCT) and MMT patients over the period 2012–2014 reported positive enhancement in the knowledge, attitudes, and practices of MMT patients in urban settings towards HIV [15]. Similarly, in a 2013 study conducted by Nguyen et al., MMT was found to produce beneficial outcomes in HIV treatment [16], while a cross-sectional study also conducted in 2013 indicated that MMT could increase HIV testing uptake among drug users [17]. Meanwhile, MMT services have drastically increased since 2008 [18], now reaching 46,000 PWID and covering 251 treatment centers [19]. MMT is provided free-of-charge by the Government for those who enrolled (enrollment is voluntary, and the patient is required to attend the clinic every day for their dosage, which has to be taken at the clinic while a health staff member is present) [20]. Adherence to treatment has been found to be sub-optimal, as a study on mountainous MMT patients conducted by Nguyen et al. indicated that only 34.4% of respondents reported optimal adherence [21]. Meanwhile, Hoang et al. found the non-adherence rate to be increasing overtime in MMT patients in urban areas [22]. Simultaneously, HIV services have been expanded to include 364 centers [19,23]. However, there have been concerns about the challenges—particularly the difficulties in accessing services due to high treatment costs—that the HIV-infected patients and the Vietnamese government would have to face as international funding (i.e., the main funding source for HIV and MMT services in the country) is decreasing [24]. As a result, maximizing services and enhancing the efficiency of HIV prevention are of even more critical importance for the battle against HIV infection.

In addition, we have noticed a lack of studies focusing on the effectiveness of MMT on PWID and other populations vulnerable to HIV risks in the mountainous regions of Vietnam. The main reason for this literature gap may be the fact that attempts to conduct studies in mountainous regions are often faced with obstacles; for instance, the clinics that can potentially be used as study settings are often in remote areas and understaffed, the prospective participants live far from clinics and usually have low levels of education and willingness to respond to complex questions, which in turn lead to difficulties in obtaining data and monitoring respondents. Nonetheless, understanding how MMT benefits risk prevention towards HIV in mountainous areas is crucial in ensuring the effectiveness of nation-wide scale-up of the MMT program, especially since these areas may hold the biggest challenges in MMT implementation. The PWID in the mountainous areas of Northern Vietnam have been reported to account for over 30% of the total PWID in Vietnam, but the majority of these patients live remote from health facilities in general and MMT clinics in particular, indicating accessibility barriers [25]. A study by Toan et al. (2002) found that people in these regions experienced a significant inequality in the services received compared to those in urban areas [26]. Moreover, the natives of mountainous provinces have also been found to have the culture of smoking opium and planting poppies, which may increase the likelihood of relapse and concurrent drug use among those enrolled in MMT. The complex nature of border regions—possibly inhabited by a number of drug criminals in hiding or who are managing drug transportation and distribution networks—add to the severity of the drug problem and hardship of MMT implementation [27]. Thus, this study aimed to explore the potential changes in the drug- and HIV-related risk behaviors of MMT users in the mountainous provinces of Northern Vietnam, as well as their associated factors.

## 2. Materials and Methods

### 2.1. Study Setting

We conducted a before–after (pre–post) study with no control group from December 2014 to December 2015 in six clinics, including Provincial AIDS Centers in Dien Bien, Lai Chau, and Yen Bai City; and District Health Centers in Thanh Xuong, Phong Tho, and Nghia Lo, which satisfied the following criteria: (1) Placed in Dien Bien, Lai Chau, and Yen Bai provinces: (2) Currently providing MMT services; (3) Covering the provincial or district levels of the Vietnamese health system.

A convenience sampling technique was adopted to recruit participants for the study. Selected participants were those who satisfied the following criteria: (1) Participating in an MMT service at one of the clinics included in the study (within 3 months) at the time of the study in 2014; (2) Agreeing to participate in research by giving written consent; (3) Having good physical and mental health to participate in the study; and (4) Having used the MMT service for 12 months. MMT patients were invited to enroll in the study when they initiated the treatment and after 12th-month of the MMT program. There were 300 patients participating in the MMT program at baseline, and 244 patients remaining after 12 months of treatment (81.3%). The 56 participants who were no longer attending their respective clinics dropped out of the study.

### 2.2. Measures and Instruments

Data were collected using face-to-face interviews with a structured questionnaire. The interviews were conducted by post-graduate students from Hanoi Medical University who had prior training in conducting community surveys. Information regarding participant socio-demographic characteristics as well as self-reported drug use status, alcohol use, smoking, and sexual behaviors was collected. This study utilized simple measures of outcomes in consideration of resource constraints (shortage of staff, limited time and difficulties in recruiting, obtaining data from and monitoring participants (due to geographical barriers, low participant education level, and low participant willingness to respond to complex questions). Nonetheless, the measures used were in line with comparable research conducted on the potential benefits of MMT implementation in Vietnam [28] and on the MMT-enrolled population in Vietnamese mountainous areas [29,30,31].

#### 2.2.1. Socio-Demographic Status

Socio-demographic characteristics of participants such as age, ethnicity, education, marital status, and occupation were also collected.

#### 2.2.2. Drug Use Behaviors

Drug use behaviors included: (1) Using drugs (whether used or not); (2) Drug injection (whether conducted injection or not), and (3) Frequency of substance use per day in the past 30 days.

#### 2.2.3. Alcohol Use and Smoking Behaviors

Alcohol use and smoking behaviors included: (1) Currently smoke; (2) Using alcohol; (3) Frequency of alcohol use, and (4) Average alcohol consumption, all within the past 30 days.

#### 2.2.4. Sexual Behaviors

Sexual behaviors included: (1) Using condoms the last time they had sex; (2) Had sex with female sex workers; (3) Using condoms when having sex with female sex workers in the past month; (4) Had sex with female sex workers who inject drugs; (5) Had sex with male partner(s); and (6) Spouse/partner injected drugs within the last 30 days.

### 2.3. Statistical Analysis

We described the frequency (n) and prevalence (%) of variables at baseline and in follow-up data. The difference between before and after MMT intervention was assessed using Chi-square and Fisher's exact test. Statistical significance was set at *p* < 0.05. Effect size (ES), which indicates the magnitude of differences, was calculated to identify the effect of MMT programs. In this study, we applied the following formula to calculate Cramér's V as representing ES for categorical outcomes with more than two levels [32]:

$$\emptyset_{c}=\;\sqrt{\frac{X^{2}}{N\left(k-1\right)}}$$

ES was classified into four categories: very small, small, medium, and large effect sizes, equating to the values 0.01, 0.1, 0.3, and 0.5, respectively [32]. The generalized estimation equation was used to find the factors associated with respondent substance use and sexual risk behavior. STATA version 12.0 (StataCorp LP, College Station, Texas, USA) was used to analyze data.

### 2.4. Ethical Approval

This study protocol was considered and obtained approval by the Scientific Research Committee of the Vietnam Authority of HIV/AIDS Control (Ethical code number 189/QD-AIDS dated 10 October 2014 and number 127/QD-AIDS dated 23 July 2015). We asked participants to provide written informed consent before starting interviews. Participants could withdraw from the study at any time. Patient information was coded and only used for research purposes.

## 3. Results

The socio-demographic characteristics of participants are described in Table 1. A total of 300 patients enrolled in the study, and 244 were taking MMT daily at their respective clinics for at least 12 months. The age groups of 30–39 and 40–49 accounted for the highest proportions of participants (36.0% and 35.7% at baseline, 35.3% and 38.9% at follow-up, respectively). Most of the participants were of the Kinh ethnic group (56.6% at follow up), had less than high school education (57.0%), and were living with a spouse/partner (58%). Freelancing was the most popular occupation (83% at baseline and 45.9% at follow-up).

Table 2 presents the drug use changes among MMT patients at the start and after one year of treatment. Significantly more participants were not using drugs after one year (83.5% vs. 63.5%), with an overall change effect size of 0.223. Meanwhile, at 12 months, 87.3% of patients reported not injecting drugs compared to 76.4% at baseline, with an effect size of 0.142. Significantly fewer participants had used drugs more than once a day over the past thirty days at the after-12-month time point (17.4% vs. 6.3% with the effect size of 0.166).

Alcohol use and smoking behaviors among the participants are illustrated in Table 3. Slightly fewer participants reported smoking after 12 months (82.2% and 86.1%) and alcohol use increased slightly from 55.1% to 62.2% after 12 months; neither change was statistically significant. The proportion of participants reporting only having alcohol 1–2 times/month at baseline were 21.6%, compared to 29% after 12 months, while the number drinking > 2 times per day fell from 25.4% to 18%. The percentage of respondents reporting drinking 3–4 times/week more than doubled (6% versus 12.7%). There was a significant change in the average consumption of alcohol, with the proportion of participants who drank > 500 mL/day increasing from 3.1% to 8.5%.

Table 4 shows the sexual behaviors of MMT patients at baseline and after 12 months. No statistically significant change was found following the one year of treatment. The percentage of participants who reported using condoms the last time they had sex was 51.3% and 51.4% for baseline and after 12 months, respectively. The minority of participants at both time points had sex with female sex workers (16.2% and 19.3%), while of those that did only 36.8% and 40% reported using condoms.

Factors associated with substance abuse and sexual risk behavior among participants are presented in Table 5. Respondents who were older (compared to being under 30 years old), of Thai ethnicity (compared to Kinh ethnicity), had high school education, worked as farmers or other jobs, and had been using drugs for longer were significantly less likely to use drugs following treatment. Those of Thai ethnicity, having at least high school education, living with a spouse/partner or being divorced/widowed, working as a freelancer, and having longer drug use history were found to have significantly higher possibility of exercising sexual risk behaviors after one-year treatment.

## 4. Discussion

To our knowledge, this study is one of a few that explore the risk behaviors of MMT patients in the mountainous areas of Vietnam with regard to before and after 12 months of engaging in MMT service. In general, it was found that the majority of MMT patients were also smokers and frequent alcohol consumers who did not display significant sex-related risk behaviors. A significant reduction was found in drug use—especially in drug injection—following a 12-month MMT program, while smoking, alcohol assumption, and sex-related behaviors showed no significant changes. In addition, the socio-economic characteristics ethnicity, education, occupation, and drug use history were found to influence the possibility of engaging in drug use and/or sexual risk behavior following treatment.

The findings of this study on drug use after 12 months of enrolment in an MMT program add to the literature supporting the effectiveness of MMT in reducing drug-related risk behaviors [33,34]. In a review of data from the six countries which reportedly account for over half of PWID globally, increased MMT services were found to be associated with decreased use of other drugs and reduced HIV risk [35]. Studies in China, which has a significant drug-using population, have shown that drug use and related risks to HIV decrease substantially upon MMT initiation—particularly in the first 6 months [7,8]. In Vietnam, it was also found in a recent study that longer engagement in MMT was associated with a lower likelihood of using other illicit drugs [36]. However, as the reduction effect found in our study was small, it is suggested that the specific context in which MMT services were conducted (e.g., in our case the mountainous regions where patients were of different ethnicity and had relatively lower level of education as compared to urban areas) would play a significant role in determining the effectiveness of an MMT program. The lack of studies on drug users and HIV-positive patients in these remote areas not only neglects a vulnerable population, but also potentially undermines the benefits of MMT scale-up.

The high prevalence of smoking and alcohol use among MMT patients found in this study is in line with the existing literature. Previous studies conducted in developed countries have indicated that cigarette use and alcohol consumption prevalence could be as high as 94% and 49%, respectively, among MMT patients [37]. Within the context of Vietnam, a study on people enrolled in MMT programs in urban and rural areas of Vietnam showed that 87.2% of participants were current smokers, while 29.6% were engaged in alcohol drinking [38]. Besides, similar indecisive results regarding the change of smoking and drinking behaviors among MMT patients following MMT program initiation and follow-up have been found in other studies. Do et al. discovered that smoking increased when the patients started with the program and decreased along with the program duration [38]. Meanwhile, changes in alcohol consumption among MMT patients vary, with some studies reporting increases and some decreases or no change [39]. A study in China indicated that the duration of MMT was negatively associated with the amount of alcohol being consumed [40]. Nonetheless, it has long been argued that MMT patients have a tendency to use smoking and alcohol consumption as a kind of supplemental substance—possibly to increase the pleasant feeling, to reduce the discomfort or withdrawal symptoms [37]. Thus, efforts should be given to interventions seeking to address drinking and smoking problems among these patients, perhaps by implementing similar programs used for the general population.

In addition, although most participants did not engage in risky sex-related behaviors, only half of them reported the use of condoms while having sex. Previous studies have also found relatively low condom use among drug users in Vietnam [41,42,43]. With regard to MMT patients in mountainous provinces, condom use in the last 12 months was found to be the range of 28% to 44.1% [31]. Since MMT patients are already at higher risk of HIV infection, unprotected sex among them may be particularly problematic. This signifies the importance of having solutions to improve condom use and safe sex behaviors in general among MMT patients, in accordance with efforts to enhance the effectiveness of MMT programs, so as to reduce overall HIV risk.

Overall, this study provides insights into the effects of MMT on the risk behaviors of patients living in mountainous regions—a relatively under-researched area. The findings can serve as a reference point for policy makers and service providers when developing strategies for MMT implementation on national and regional levels. The need for the scaling-up of MMT programs as well as for providing additional counseling services targeting alcohol and cigarette use and HIV testing and counseling services at MMT clinics—which has been proved elsewhere [17,44,45,46]—is further supported by this study. It is also suggested that more resources should be allocated to MMT implementation in mountainous areas, given the inherent challenges in these areas. Close collaboration between regional health authorities, border authorities, and communities in promoting the reduction of risk behaviors—especially opium use, unsafe sex, and involvement in drug transportation and distribution activities—is also advised.

The most significant limitation of this study is the small sample size, as only 244 participants were included in the 12-month data. Over the course of the 12-month period, 56 patients withdrew from the study, and the exclusion of data regarding these dropouts may skew our results toward positive, improved risk behavior following MMT adherence. We suggest that further research on this topic have more frequent follow-ups with patients in order to reduce the drop-out rate and obtain less potential bias in results. The convenient sampling technique adopted when recruiting participants may also have undermined the representativeness of our study. Although efforts were made to ensure the participants’ anonymity, the lack of a control group and the reliance on self-reported data may have introduced a social desirability bias into our results due to cultural factors. Besides, despite having included participants across six provincial and district centers in an attempt to have a relatively good representative sample of drug users in the area of interest, using MMT patients to examine risk behaviors regarding HIV may have caused some skewing of the data towards lower risk. This is because these patients voluntarily came forward to better their health and therefore may have possibly already engaged in less risky behavior overall. Further studies accessing the hidden drug-using population in order to fully comprehend risk behavior are thus called for.

## 5. Conclusions

Our data indicate that after 12 months of MMT, drug-related risk behavior for HIV was significantly reduced, but that alcohol-related and sex-related risk factors remained an issue. This information identifies aspects of MMT services that are lacking and areas where education and patient management are needed in order to reduce HIV risk even further. Given that HIV is a significant risk among PWID in Vietnam (especially in mountainous areas where health service usage is lower), the positive effects of MMT in lowering this risk are promising.

## Figures and Tables

**Table 1:** Participant socio-demographic characteristics.

Characteristics	Baseline		Follow-Up		*p*-Value
	*N*	%	*n*	%	
**Total**	300	100.0	244	100.0	
**Age group**					0.84
<30	51	17.0	36	14.8	
30–39	108	36.0	86	35.3	
40–49	107	35.7	95	38.9	
≥50	34	11.3	27	11.1	
**Ethnic**					0.88
Kinh	176	58.7	138	56.6	
Thai	81	27.0	70	28.7	
Others	43	14.3	36	14.8	
**Education**					0.96
< High school	168	56.2	139	57.0	
High school	105	35.1	83	34.0	
> High school	26	8.7	22	9.0	
**Marital status**					0.97
Single	79	26.3	65	26.8	
Living with spouse/partner	177	59.0	141	58.0	
Divorced, widowed	44	14.7	37	15.2	
**Employment**					0.25
Unemployed	24	8.0	29	11.9	
Freelancer	249	83.0	112	45.9	
Farmer	9	3.0	72	29.5	
Others	18	6.0	31	12.7	

**Table 2:** Patient drug use characteristics.

Characteristics	Baseline		Follow-Up		*p*-Value	Effect Size
	*n*	%	*n*	%		
**Drugs use**						
No	155	63.8	197	83.5	<0.01	0.223
Yes	88	36.2	39	16.5		
**Drug injection**						
No	184	76.4	206	87.3	<0.01	0.142
Yes	57	23.7	30	12.7		
**Frequency of substance use per day in the last 30 days**						
1 time	71	82.6	60	93.8	0.04	0.166
>1 time	15	17.4	4	6.3		
**Drug rehabilitation**						
No	89	29.7	68	27.9		0.020
Yes	211	70.3	176	72.1	0.65	0.020
	**Mean**	**SD**	**Mean**	**SD**		
**Length of drug use (years)**	9.3	6.6	9.8	6.7	0.30	0.077

**Table 3:** Changes in alcohol use and smoking among methadone maintenance treatment patients.

Characteristics	Baseline		Follow-Up		*p*-Value	Effect Size
	*n*	%	*n*	%		
**Currently smoke**						
No	34	13.9	43	17.8	0.24	0.053
Yes	210	86.1	198	82.2		
**Alcohol use**						
No	109	44.9	91	37.8	0.11	0.072
Yes	134	55.1	150	62.2		
**The frequency of alcohol use**						
1–2 times/month	29	21.6	42	28.0	0.14	0.156
1–2 times/week	29	21.6	27	18.0		
3–4 times/week	8	6.0	19	12.7		
1 time/day	34	25.4	35	23.3		
>2 times/day	34	25.4	27	18.0		
**Average alcohol consumption**						
Sometimes	46	35.9	33	23.2	0.04	0.193
25 mL/day	34	26.6	37	26.1		
50–100 mL/day	24	18.8	41	28.9		
250 mL/day	20	15.6	19	13.4		
≥500 mL/day	4	3.1	12	8.5		

**Table 4:** Changes in sexual behaviors among methadone maintenance treatment patients.

Characteristics	Baseline	Follow-Up			*p*-Value	Effect Size
	*n*	%	*n*	%		
**Using condoms in the last sex**						
No	114	48.7	106	48.6	0.98	0.001
Yes	120	51.3	112	51.4		
**Had sex with female sex workers**						
No	197	83.8	176	80.7	0.39	0.041
Yes	38	16.2	42	19.3		
**Using condoms when having sex with female sex workers in the last month**						
No	12	63.2	9	60.0	0.85	0.032
Yes	7	36.8	6	40.0		
**Had sex with female sex workers who inject drugs**						
No	36	87.8	42	93.3	0.31	0.095
Yes	5	12.2	3	6.7		
**Had sex with a male partner**						
No	231	99.1	216	99.1	0.66	0.003
Yes	2	0.9	2	0.9		
**Spouse/partner injected drugs**						
No	208	98.1	204	98.6	0.51	0.017
Yes	4	1.9	3	1.5		

**Table 5:** Factors associated with substance abuse and sexual risk behavior among respondents.

Characteristics	Drug Use		Sexual Risk Behavior	
	OR	95% CI	OR	95% CI
**Age Group (<30—REF)**				
30-39	0.80	(0.40–1.63)	1.15	(0.55–2.43)
40-49	0.21 ***	(0.09–0.49)	0.90	(0.39–2.08)
≥50	0.36 *	(0.12–1.08)	1.07	(0.37–3.05)
**Ethnic (Kinh—REF)**				
Thai	0.53 *	(0.27–1.04)	0.56 *	(0.29–1.07)
Others	1.05	(0.49–2.27)	0.79	(0.38–1.64)
**Education (<High school—REF)**				
High school	0.61 *	(0.34–1.08)	1.61 *	(0.92–2.82)
>High school	0.85	(0.35–2.06)	2.73 **	(1.09–6.85)
**Marital status (Single—REF)**				
Living with spouse/partner	1.06	(0.56–1.98)	7.68 ***	(3.72–15.86)
Divorced, widowed	0.88	(0.38–2.02)	2.41 **	(1.03–5.61)
**Employment (Unemployed—REF)**				
Freelancer	0.64	(0.29–1.41)	0.50 *	(0.22–1.12)
Farmer	0.35 **	(0.14–0.92)	0.74	(0.29–1.89)
Others	0.37 *	(0.14–1.01)	0.71	(0.27–1.89)
**Drug rehabilitation (No vs. Yes)**	0.66	(0.37–1.20)	0.87	(0.50–1.50)
**Length of drug use (Years)**	1.05 **	(1.01–1.10)	0.95 **	(0.91–0.99)

*** *p* < 0.01, ** *p* < 0.05, * *p* <0.1.
